# ACE2 Is a Prognostic Biomarker and Associated with Immune Infiltration in Kidney Renal Clear Cell Carcinoma: Implication for COVID-19

**DOI:** 10.1155/2021/8847307

**Published:** 2021-01-28

**Authors:** Xinhao Niu, Zhe Zhu, Enming Shao, Juan Bao

**Affiliations:** Department of Urinary Surgery, The Shanghai Public Health Clinical Center, Fudan University, Shanghai 201508, China

## Abstract

**Background:**

KIRC is one of the most common cancers with a poor prognosis. ACE2 was involved in tumor angiogenesis and progression in many malignancies. The role of ACE2 in KIRC is still ambiguous.

**Methods:**

Various bioinformatics analysis tools were investigated to evaluate the prognostic value of ACE2 and its association with immune infiltration in KIRC.

**Results:**

ACE2 was shown to be downregulated in KIRC at the mRNA and protein level. Low expression of ACE2 protein in KIRC patients was observed in subgroup analyses based on gender, age, weight, tumor grade, and cancer stage. Upregulation of ACE2 in KIRC was associated with a favorable prognosis. ACE2 mRNA expression showed a positive correlation with the abundance of immune cells (B cells, CD8+ T cells, macrophages, neutrophils, and dendritic cells) and the level of immune markers of different immune cells in KIRC. ACE2 expression could affect, in part, the immune infiltration and the advanced cancer stage. Moreover, enrichment analysis revealed that ACE2 in KIRC were mainly involved in translation factor activity, immunoglobulin binding, metabolic pathways, transcriptional misregulation in cancerous cells, cell cycle, and ribosomal activity. Several ACE2-associated kinases, miRNA, and transcription factor targets in KIRC were also identified.

**Conclusion:**

ACE2 was downregulated in KIRC and served as a prognostic biomarker. It was also shown to be associated with immune infiltration.

## 1. Introduction

Kidney cancer is one of the most common malignances globally, accounting for about 4.5% of all newly diagnosed malignances [1]. It is anticipated that 73,750 people would be newly diagnosed with kidney cancer and 14,830 patients are likely to die because of the disease in the USA in 2020 [2]. The most common subtype of renal cancer is kidney renal clear cell carcinoma (KIRC), which makes up over 70% of kidney cancers [3]. Surgery excision remains the primary therapy for KIRC due to the growing resistance to radiotherapy and chemotherapy [4]. Much worse, the prognosis of KIRC patients tends to be poor, especially for patients in an advanced stage. The five-year overall survival rate of stage IV patients is less than 10% [5]. Previous studies have revealed that immune infiltration is significantly linked to the survival of KIRC patients. [6, 7]. Immunotherapy has been suggested as the treatment for metastatic KIRC [8, 9]. Therefore, clarifying the role of immune infiltration in KIRC and identifying immune-associated markers for the prognosis for KIRC are particularly necessary.

Angiotensin converting enzyme 2 (ACE2) is a member of the renin angiotensin system (RAS) whose open reading framework encodes an 805-amino-acid polypeptide [10]. Increasing evidence indicates a significant function of ACE2 in the tumor angiogenesis and its progression in many cancers, such as thyroid carcinoma, breast carcinoma, and lung adenocarcinoma [11–13]. ACE2 has also been suggested as a biomarker for many diseases, including squamous cell/adenosquamous carcinoma, endometrial carcinoma, and hypertension [10, 14, 15]. However, limited studies have clarified the function of ACE2 in immune infiltration and its role in the prognosis in KIRC.

Coronavirus disease 2019 (COVID-19), caused by the novel coronavirus severe acute respiratory syndrome coronavirus 2 (SARS-CoV-2), was initially found in Wuhan of China since December 2019 [16, 17]. It is well known that the functional host receptor of SARS-CoV-2 is ACE2 [18, 19]. Over 10 million peoples were diagnosed with COVID-19 and over 520000 peoples died of this disease globally until July 1, 2020. As we have seen, the prognosis of COVID-19 patients with KIRC remains ambiguous.

Therefore, our study was performed to detect ACE2 levels and the prognostic value in patients with KIRC. The function of ACE2 in immune infiltration in KIRC was also clarified. Our results may provide additional evidence regarding the role of ACE2 and immune infiltration in patients with KIRC.

## 2. Materials and Methods

### 2.1. ACE2 Expression Analysis in the Oncomine™, UALCAN, and Human Protein Atlas

ACE2 expression in KIRC was identified in the Oncomine (https://www.oncomine.org/), UALCAN (http://ualcan.path.uab.edu/cgi-bin/ualcan-res.pl), and Human Protein Atlas (https://www.proteinatlas.org/). ACE2 mRNA levels in various malignances, including KIRC, were determined with the Oncomine database and the threshold was set to the *P* value = 0.05 and fold change (FC) = 2, as well as gene ranking = top 10% [20]. In order to further detect the ACE2 protein expression in KIRC, we then used UALCAN and Human Protein Atlas. Based on data from Clinical Proteomic Tumor Analysis Consortium (CPTAC), UALCAN could be also used to detect the ACE2 protein expression in various subtribes of patients with KIRC [21]. The Human Protein Atlas is a program designed to map all of the human proteins in the cells, tissues, and organs [22]. Immunohistochemical staining of ACE2 in KIRC was obtained from the Human Protein Atlas.

### 2.2. Prognosis Analysis in GEPIA and Kaplan–Meier (KM) Plotter

In order to evaluate the significance of ACE2 level in the prognosis of KIRC, GEPIA (http://gepia.cancer-pku.cn/) [23], OSkirc (http://bioinfo.henu.edu.cn/KIRC/KIRCList.jsp) [24], and KM plotter databases (https://kmplot.com/) were applied separately. The median value of ACE2 expression was utilized to identify high/low ACE2 expression patients and the *P* value was set as 0.05. In Meier plotter, subgroup prognosis analysis based on different clinicopathologic features and immune cells in KIRC was performed using TCGA KIRC dataset.

### 2.3. TIMER for Immune Infiltrates Analysis

TIMER (https://cistrome.shinyapps.io/timer/) is a comprehensive tool providing immune infiltrates analysis across TCGA tumors [25]. Immune cell infiltration and immune biomarker expression were correlated with ACE2 and were evaluated with Spearman's correlation analysis using the TCGA KIRC dataset. The immune cells included were B cells, CD4+ T cells, CD8+ T cells, neutrophils, macrophages, and dendritic cells. Immune biomarkers were excluded because they have already been described in previous studies [26–28].

### 2.4. cBioPortal for Genetic Alteration Analysis

cBioPortal (http://www.cbioportal.org) is a TCGA visual tool used to perform genome analysis [29]. We analyzed ACE2 genetic alteration in KIRC with the threshold as ±2.0 in mRNA expression z-scores (RNASeq V2 RSEM) and protein expression z-scores (RPPA).

### 2.5. LinkedOmics for Enrichment Analysis

In order to verify the ACE2-associated functions in KIRC, LinkedOmics (http://www.linkedomics.org/), a comprehensive tool for multiomics analysis, was used [30]. A Pearson correlation test was used to explore genes that are linked to ACE2 in KIRC, while gene set enrichment analysis (GSEA) was performed for the enrichment analyses (GO and KEGG pathways), and ACE2-associated targets (kinase, miRNA, and transcription factor) were obtained with GSEA. These analyses were carried out using the TCGA KIRC dataset, with a *P* value < 0.05.

## 3. Results

### 3.1. The Expression of ACE2 in KIRC

**Figure 1:**
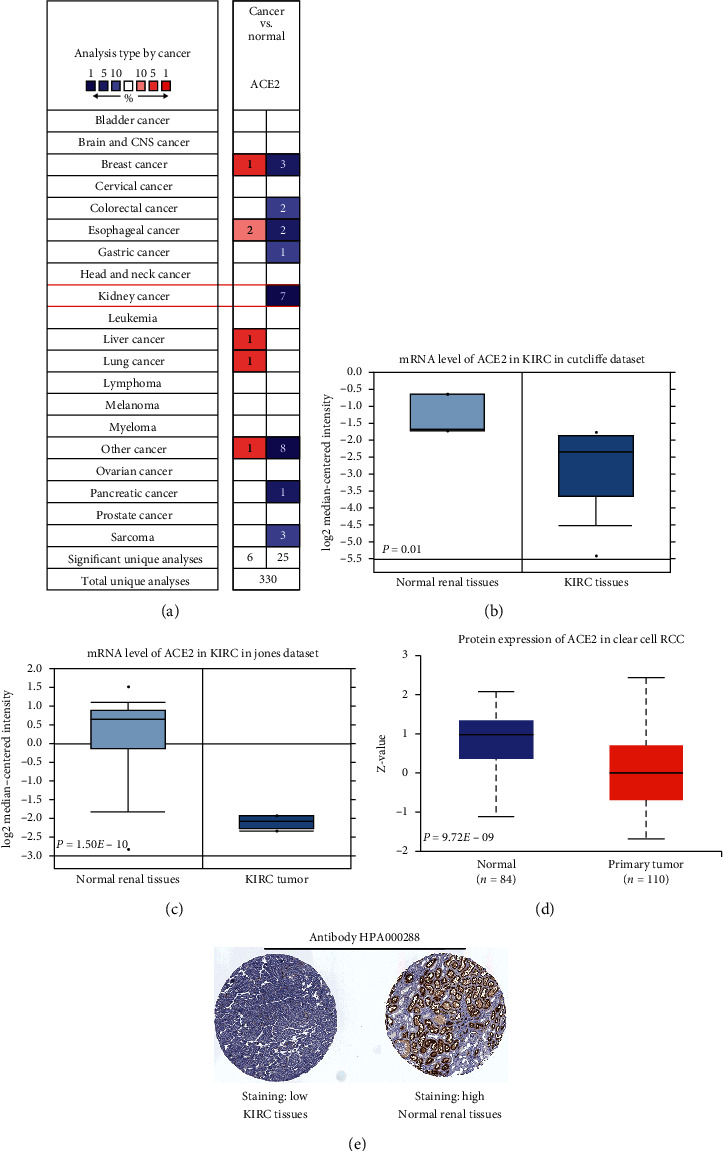
The level of ACE2 in KIRC. (a) Upregulation or downregulation of ACE2 in different types of cancers, including KIRC, compared to the different types of normal tissues. (Oncomine). ((b), (c)) Plot showing ACE2 mRNA expression in KIRC and normal tissues in the dataset from Oncomine. (d) Plot showing ACE2 protein expression in KIRC and normal tissues in the dataset from UALCAN. (e) Immunohistochemical staining showing the protein level of ACE2 in KIRC and normal tissue (the Human Protein Atlas).

We initially detected the mRNA and protein expression of ACE2 in KIRC in Oncomine, UALCAN, and Human Protein Atlas. According to the data from Oncomine, ACE2 mRNA expression was dramatically reduced in KIRC when compared with normal kidney tissues (Figures 1(a)–1(c)). A gene expression profile also revealed that ACE2 mRNA expression was reduced in KIRC when compared with normal kidney tissues, with an FC of −2.843 as well as a *P* value of 0.01 (Figure 1(b)) [31]. Another study indicated that ACE2 mRNA is expressed 5.131 times more in renal tissues than in KIRC tissues (Figure 1(c), *P*=1.50*E* − 10) [32]. In order to further verify these results, we decided to use the CPTAC dataset to observe ACE2 protein expression. As expected, the results demonstrated a downwards regulation of ACE2 protein expression in KIRC when compared with normal kidney tissues (Figure 1(d)). ACE2 protein expression was detected with staining and the expression data from the Human Protein Atlas. Interestingly, the immunohistochemical staining map suggested a low protein expression of ACE2 in KIRC tissues with a high protein expression of ACE2 in normal kidney tissues (Figure 1(e)).

**Figure 2:**
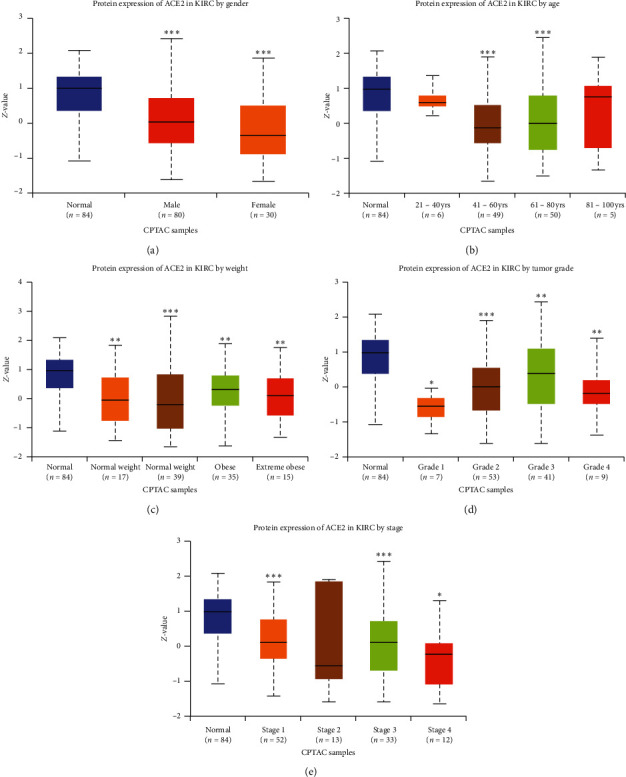
The protein expression of ACE2 in subgroups of patients with KIRC (UALCAN). (a) ACE2 protein expression in normal and KIRC (male or female) samples. (b) ACE2 protein expression in normal and KIRC (21–40, 41–60, 61–80, or 81–100 years old) samples. (c) ACE2 protein expression in normal and KIRC (normal weight, extreme weight, obese, or extreme obese) samples. (d) ACE2 protein expression in normal and KIRC (grade 1, 2, 3, or 4) samples. (e) ACE2 protein expression in normal and KIRC (Stage 1, 2, 3, or 4) samples. Data are mean ± SE. *∗P* < 0.05; *∗∗P* < 0.01; *∗∗∗P* < 0.001.

However, we evaluated ACE2 protein expression in various subtribes of patients with KIRC. The results are shown in Figure 2. This indicates a low expression of the ACE2 protein in KIRC patients in the subtribes analyses based on gender, age, weight, tumor grade, and cancer stage. Therefore, ACE2 was downregulated in KIRC and may be involved in tumor progression.

### 3.2. ACE2 Could Serve as a Prognostic Biomarker in KIRC

**Figure 3:**
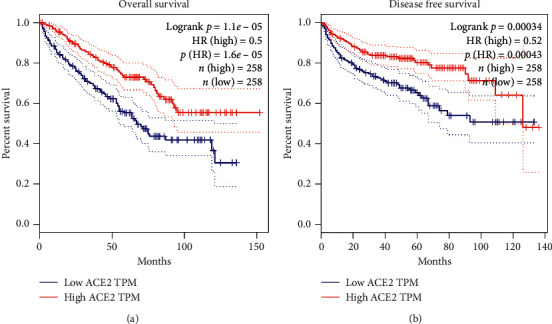
ACE2 served as a biomarker in KIRC. (a) High ACE2 expression in KIRC was associated with a favorable overall survival (GEPIA). (b) High ACE2 expression in KIRC was associated with a favorable disease-free survival (GEPIA). The median value of ACE2 expression was utilized to identify high/low ACE2 expression patients.

A Kaplan–Meier curve was applied using TCGA KIRC and GSE29609 datasets for prognosis analysis. KIRC patients with a high level of ACE2 expression were strongly correlated with better overall survival (OS) (Figure 3(a), logrank *P*=1.1*e* − 05) and disease-free survival (DFS) rates (Figure 3(b), logrank *P*=0.000034). Thus, ACE2 could potentially serve as a prognostic biomarker in KIRC patients.

The correlation between ACE2 expression and clinical characteristics of KIRC patients in the Kaplan–Meier plot was also explored to see how ACE2 expression affects the prognosis of patients with KIRC. As shown in Table 1, increasing levels of ACE2 were linked to better prognosis in male and female patients and high/low mutation burden patients (all *P* < 0.05). Moreover, an increased expression level of ACE2 was linked to better prognosis in tumor grades 2 to 4 of KIRC patients. However, there is not enough data about KIRC patients in tumor grade 1 to perform the same analysis. Specifically, the increasing level of ACE2 was linked to better prognosis in cancers in stages 2 to 4 of KIRC patients (All *P* < 0.05) but was not linked to better prognosis in cancer stage 1 patients (HR = 0.57, *P*=0.069, Table 1). These data demonstrate that ACE2 expression could affect the prognosis of KIRC patients with advanced cancer stage.

### 3.3. ACE2 Was Associated with Tumor Immune Infiltration in KIRC

**Figure 4:**
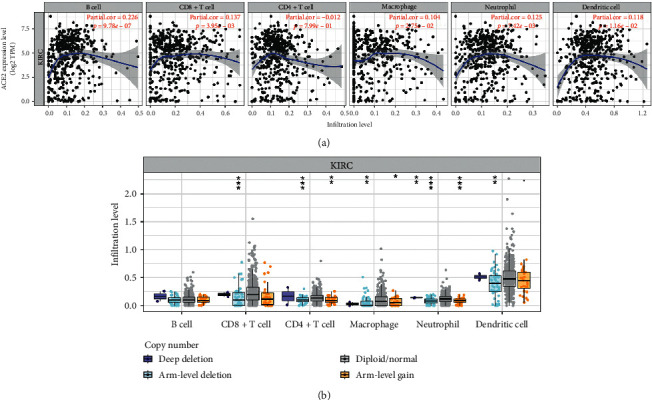
The correlation between ACE2 and immune infiltration (TIMER). (a) The correlation between ACE2 expression, the abundance of CD8+ T cells, CD4+ T cells, macrophages, neutrophils, and dendritic cells. (b) The correlation between SCNA of ACE2 and immune-cell infiltration. SCNA, somatic copy number alterations; *∗P* < 0.05; *∗∗P* < 0.01; *∗∗∗P* < 0.001.

Previous studies have highlighted the significance of the tumor immune infiltration in the prognosis of renal cancer [6, 33]. Therefore, we evaluated the correlation between ACE2 mRNA expression and immune infiltration in KIRC using the TIMER database. Interestingly, ACE2 mRNA expression showed a positive link to the abundance of B cells (*P*=9.78*e* − 07), CD8+ T cells (*P*=0.00395), macrophages (*P*=0.0275), neutrophils (*P*=0.00742), and dendritic cells (*P*=0.0116) (Figure 4(a)). Conversely, the copy number alteration of ACE2 could inhibit immune infiltration (Figure 4(b)).

We further investigated if the expression of ACE2 was associated with immune markers of different immune cells in KIRC. As expected, a significant correlation was obtained between the expression of ACE2, and most of the immune markers in KIRC after tumor purity modulation were performed (Table 2). Specifically, ACE2 was strongly linked to CD8A and CD8B (CD8+ T cell), CD19 and CD79A (B cell), CD86, and CD115 (monocyte), as well as CCL2 and CD68 (TAM). ACE2 was also positively linked to all markers of M1 macrophage (INOS, IRF5, and PTGS2). Moreover, ACE2 levels showed a positive association with most markers of natural killer cell (KIR2DL1, KIR2DL3, KIR2DL4, KIR3DL1, and KIR3DL2), Dendritic cell (HLA-DPB1, HLA-DQB1, HLA-DRA, HLA-DPA1, CD1 C, and NRP1), and Th2 (GATA3, STAT6, and STAT5A). Similarly, ACE2 in KIRC showed a positive correlation with STAT3 in Th17, FOXP3, STAT5B, and TGFB1 in Treg, as well as TIM-3 in T cell exhaustion (Table 2). Taken together, ACE2 was associated with tumor immune infiltration in KIRC, and ACE2 may play a vital role in immune escape in the KIRC microenvironment.

### 3.4. Prognostic Analysis of ACE2 Expression in KIRC Based on Immune Cell Analysis

**Figure 5:**
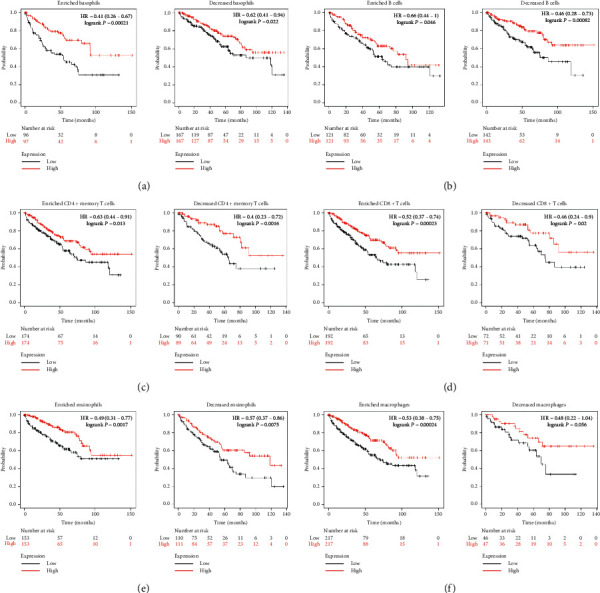
Prognostic value of ACE2 in KIRC based on immune-cell subgroups (Kaplan–Meier plotter).

**Figure 6:**
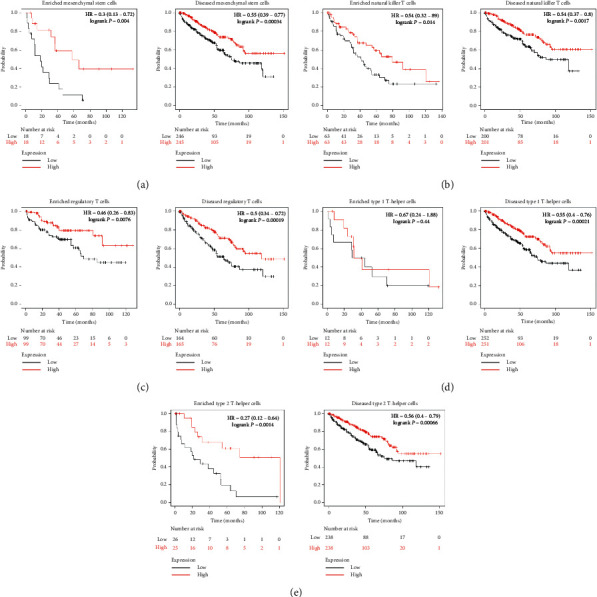
Prognostic value of ACE2 in KIRC based on immune-cells subgroup (Kaplan–Meier plotter).

The abovementioned results found that the expression levels of ACE2 were associated with favorable prognoses and immune infiltration in patients with KIRC. A prognostic analysis was performed to verify if the expression of ACE2 affects prognosis and immune infiltration in KIRC. This was based on immune cells using the Kaplan–Meier plotter. As we could see in Figure 5, high expression of ACE2 in KIRC from the cohorts of enriched/decreased basophils (Figure 5(a)), enriched/decreased B cells (Figure 5(b)), enriched/decreased CD4+ memory T cells (Figure 5(c)), enriched/decreased CD8+ T cells (Figure 5(d)), and enriched/decreased eosinophils (Figure 5(e)) were associated with favorable prognosis. Similarly, the high expression of ACE2 in KIRC from the cohorts of enriched/decreased mesenchymal stem cells (Figure 6(a)), enriched/decreased natural killer T cells (Figure 6(b)), enriched/decreased regulatory T cells (Figure 6(c)), and enriched/decreased type 2 T-helper cells (Figure 6(e)) were also linked to a better prognosis. However, the high expression of ACE2 in KIRC from the cohorts of enriched macrophages (Figure 6(f)) and decreased type 1 T-helper cells (Figure 6(d)) were associated with a favorable prognosis. However, no correlation was observed between the high expression of ACE2 and the prognosis of KIRC in decreased macrophages (Figure 6(f)) and enriched type 1 T-helper cell cohorts (Figure 6(d)). Therefore, ACE2 may affect the prognosis of patients with KIRC, in part, due to immune infiltration.

### 3.5. Genetic Alteration of ACE2 in KIRC

**Figure 7:**
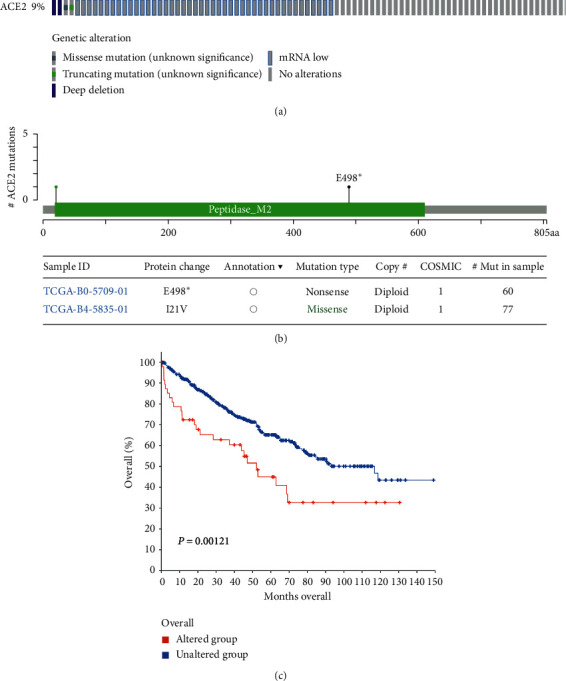
Genetic alteration of ACE2 in KIRC patients (cBioPortal). (a) OncoPrint visual summary of alteration on ACE2 in KIRC. (b) The missense mutation of ACE2 amino acids was analyzed. Color images are available online. (c) The overall survival in cases with/without ACE2 alterations.

Genomic mutations are known to be significantly linked to tumorigenesis. In our study, genetic alteration analysis of ACE2 in KIRC patient datasets revealed that a total of 9% of genetic alterations in ACE2 in KIRC and the genetic alteration form contained missense mutations, truncating mutations, deep deletions, and low mRNA (Figure 7(a)). Moreover, ACE2 mutations could lead to protein change, including E489∗ and I21 V (Figure 7(b)). Interestingly, we found that ACE2 alterations in KIRC predicted a worse overall survival rate (*P*=0.00121, Figure 7(c)). These findings suggest that an ACE2 genetic alteration may regulate tumorigenesis and its progression to KIRC, thus impacting the patients' prognosis.

### 3.6. Enrichment Analysis of ACE2 in KIRC

**Figure 8:**
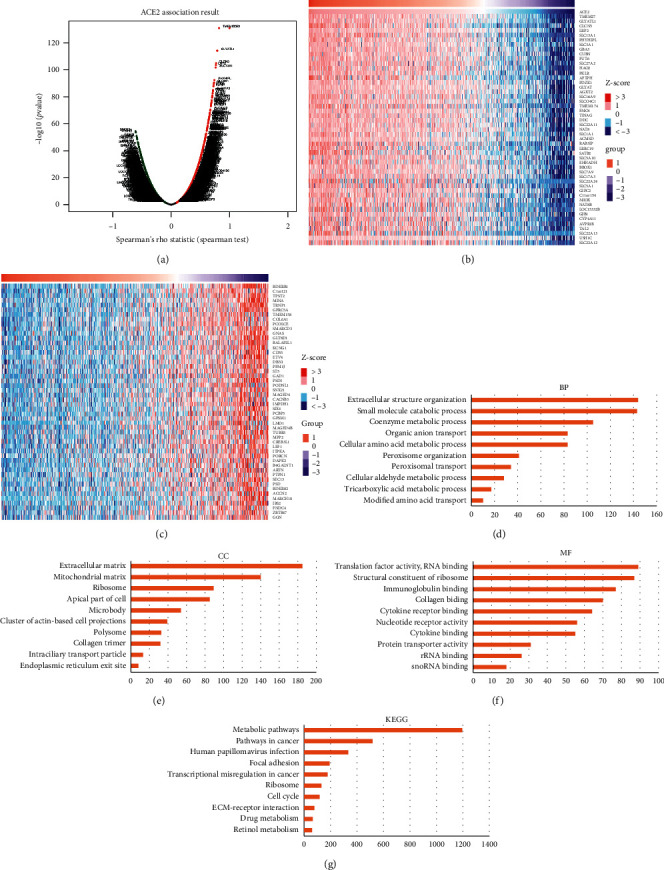
Enrichment analysis of ACE2 in KIRC (LinkedOmics). (a) The differentially expressed genes significantly correlated with ACE2 in KIRC. ((b), (c)) Heat maps showing the top 50 genes positively and negatively correlated with ACE2 in KIRC. (d) BP analysis. (e) CC analysis. (f) MF analysis. (g) KEGG pathway analysis. The analysis was performed by GSEA.

The TCGA KIRC dataset was analyzed with LinkedOmics.  Figure 8(a) shows that 3792 genes were positively linked to ACE2, and 6892 genes were negatively linked to ACE2 (false discovery rate <0.01). The top 50 significant genes that showed a positive and negative correlation with ACE2 were also obtained (Figures 8(b) and 8(c)). GSEA was performed to analyze GO enrichment analysis, which revealed that ACE2 in KIRC were mainly involved in extracellular structure organization, small molecule catabolic processes, cellular amino acid metabolic processes, translation factor activity, structural constituent of ribosomes, immunoglobulin binding, cytokine receptor binding, and RNA binding (Figure 8(d)–8(f), *P* < 0.05). Moreover, the KEGG pathway items indicate that ACE2 in KIRC was mainly in charge of metabolic pathways, pathways in cancer, focal adhesion, transcriptional misregulation in cancer cells, cell cycle, and ribosomes (Figure 8(g), *P* < 0.05).

### 3.7. ACE2-Associated Targets in KIRC

To further clarify the underlining mechanisms of how ACE2 affects tumorigenesis and the progression of KIRC, we explored ACE2-associated kinase, miRNA, and transcription factor targets in KIRC using GSEA in LinkedOmics. As a result, the top five most significant ACE2-associated kinase targets in KIRC were Kinase_LCK, Kinase_LYN, Kinase_SYK, Kinase_JAK3, and Kinase_HCK (Table 3, all *P* < 0.05), and the top five ACE2-associated miRNA targets were MIR-96 (GTGCCAA), MIR-519C, MIR-519B and MIR-519A (TGCACTT), MIR-148A, MIR-152, and MIR-148B (TGCACTG), MIR-506 (GTGCCTT), and MIR-374 (TATTATA) (Table 3, all *P* < 0.05). In the transcription factor target analysis, the results demonstrated V$IRF_Q6, V$NFKB_Q6_01, V$ELF1_Q6, V$PEA3_Q6, and V$PU1_Q6 as the ACE2-associated targets in KIRC (Table 3, all *P* < 0.05).

## 4. Discussion

ACE2, a novel identified component of RAS, could regulate the tumorigenesis and progression in cancers and serve as a biomarker for many diseases [34–36]. Moreover, increasing evidence highlights the association between ACE2, tumor microenvironment, and immune infiltration [10, 37]. However, there were limited studies that clarified the function of ACE2 in immune infiltration and the prognosis of KIRC. Therefore, our study was undertaken.

The expression analysis revealed that ACE2 was downregulated in KIRC patients at the mRNA and protein level, and a low expression of ACE2 protein in KIRC patients was obtained in the subgroup analysis. These results indicate that ACE2 may play a significant role in KIRC. Further prognosis analysis indicated that high ACE2 level in KIRC patients was linked to a favorable prognosis in both the TCGA and GEO cohorts, suggesting ACE2 could be a novel prognostic biomarker for KIRC and the prediction of a favorable outcome. ACE2 has also been suggested as a biomarker for other diseases or cancers. In thyroid carcinoma, ACE2 was employed as a biomarker and was also found to regulate tumor progression [13]. Moreover, ACE2 acted as a biomarker in chronic kidney disease and associated with higher risk for silent atherosclerosis [36].

A steady accumulation of data suggests that immune‐cell infiltration could regulate tumor progression and metastasis, thus affecting the patients' prognosis [38, 39]. In our study, we also clarified the correlation between ACE2 and immune infiltration. We found ACE2 to be positively associated with the abundance of immune cells, including B cells, CD8+ T cells, macrophages, neutrophils, and dendritic cells. Moreover, a strong correlation between the expression of ACE2 and most of the immune biomarker sets were analyzed. These immune cells or biomarkers were known to be involved in tumor progression and as a biomarker for prognosis or therapy of KIRC. Ying et al. found that tumor microenvironment B cells were associated with poor survival and reduced response to treatment [40]. Moreover, CD39+ CD8+ T cells were shown to act as prognostic biomarkers in patients with KIRC and were also used to indicate poor prognosis [41]. In our study, we also found that ACE2 may affect the prognosis of KIRC patients, in part, due to immune infiltration. However, previous studies have suggested that T-regulatory cells are correlated with the poor outcomes of patients with KIRC [6]. However, we found that ACE2 positively associated with the abundance of several immune cells. Thus, all correlations of ACE2 and infiltrating immune cells in KIRC may not be favorable. Further study should be performed to verify these results and observations.

The enrichment analysis suggested that ACE2 in KIRC were primarily involved in translation factor activity, immunoglobulin binding, metabolic pathways, transcriptional misregulation in cancer cells, cell cycle, and ribosomes. These findings are consistent with the previous study where ACE2 was associated with cell cycle transcription [42]. Misregulation of ribosome functions and the cell cycle has been linked to many diseases, in particular cancers [43]. Our study further confirmed the association of ACE2 with transcriptional misregulation and the cell cycle in patients with KIRC.

Genomic instability and mutagenesis could result in tumor genesis and progression while kinase could stabilize and repair genomic DNA [44]. In our study, several ACE2-associated kinase targets included LCK, LYN, SYK, JAK3, and HCK. Interestingly, these kinases were mainly involved in regulating genomic stability, cell cycle, ribosomal activity, and immune infiltration [45, 46]. These are consistent with the findings that ACE2 in KIRC were primarily involved in the cell cycle, ribosomal activity, and immune infiltration. Previous studies suggest that JAK3 is used as a biomarker and is associated with immune infiltration in patients with KIRC [7]. Another study found that alterations in the JAK3 pathway were involved in CD8 T cell immune deviation in RCC [47]. Therefore, ACE2 may regulate immune infiltration via JAK3 kinase. However, further research needs to be done to confirm this.

Transcriptional dysregulation and cell cycle disorder could lead to constant proliferation and abnormal cell invasion, which is the fundamental feature of cancers. Transcription factors are the key regulator of transcriptional activity and the cell cycle [48]. Our study identified several ACE2-associated transcription factor targets in KIRC, such as V$IRF_Q6, V$NFKB_Q6_01, V$ELF1_Q6, V$PEA3_Q6, and V$PU1_Q6. PEA3 could block cell cycle progression in breast cancer [49]. Moreover, PEA3 could facilitate cell invasion and metastasis in colorectal carcinoma [50]. Therefore, ACE2 may regulate the cell cycle and progression of KIRC via these transcription factors. Further studies should be performed to test this hypothesis.

## 5. Conclusion

In conclusion, our results indicate that ACE2 may be developed as a prognostic biomarker. It is associated with immune infiltration in patients with KIRC. This lays a foundation for further study of the function of ACE2 in the carcinogenesis and progression of KIRC.

## Figures and Tables

**Table 1:** Correlation of ACE2 expression and the overall survival of KIRC with different clinicopathological factors (Kaplan–Meier plotter).

Pathological parameters	Case number	Hazard radio	*P* value
Stage status			
1	398	0.57 (0.31–1.05)	0.069
2	184	0.29 (0.1–0.89)	0.021
3	332	0.34 (0.19–0.6)	0.00011
4	188	0.26 (0.15–0.45)	2.5*e*^−7^
Gender			
Female	284	0.41 (0.25–0.68)	0.00038
Male	948	0.35 (0.24–0.52)	2*e*^−8^
White	690	0.36 (0.26–0.5)	3.2*e*^−11^
Asian	8	NA	NA
Black/African-American	111	2.81 (0.59–13.37)	0.18
Tumor grade			
1	14	NA	NA
2	340	0.5 (0.28–0.92)	0.022
3	585	0.38 (0.24–0.61)	2.2*e*^−5^
4	174	0.42 (0.23–0.77)	0.0039
Mutation burden			
high	246	0.43 (0.25–0.76)	0.0027
low	437	0.34 (0.16–0.75)	0.0051

**Table 2:** Correlation analysis between ACE2 and gene biomarkers of immune cells in KIRC (TIMER).

Description	Biomarkers	None		Purity	
		Cor.	*P* value	Cor.	*P* value
CD8+ T cell	CD8A	0.0.119	∗∗	0.107	∗
	CD8B	0.115	∗∗∗	0.099	∗
T cell (general)	CD3D	0.045	0.298	0.027	0.565
	CD3E	0.064	0.14	0.048	0.302
	CD2	0.09	∗	0.077	0.0985
B cell	CD19	−0.176	∗∗∗	−0.186	∗∗∗
	CD79A	−0.12	∗∗	−0.144	∗∗
Monocyte	CD86	0.126	∗∗	0.117	∗
	CD115 (CSF1R)	0.096	∗	0.092	∗
TAM	CCL2	0.0.266	∗∗∗	0.28	∗∗∗
	CD68	0.146	∗∗∗	0.097	∗
	IL10	0.006	0.885	−0.016	0.733
M1 macrophage	INOS (NOS2)	0.2	∗∗∗	0.18	∗∗∗
	IRF5	0.229	∗∗∗	0.208	∗∗∗
	COX2 (PTGS2)	−0.219	∗∗∗	−0.205	∗∗∗
M2 macrophage	CD163	0.066	0.128	0.04	0.396
	VSIG4	−0.027	0.538	−0.061	0.188
	MS4A4A	0.019	0.668	0.002	0.973
Neutrophil	CD66b (CEACAM8)	0.071	0.0996	0.053	0.255
	CD11b (ITGAM)	0.159	∗∗∗	0.147	∗∗∗
	CCR7	−0.004	0.932	−0.039	0.403
Natural killer cell	KIR2DL1	0.17	∗∗∗	0.136	∗∗
	KIR2DL3	0.139	∗∗	0.106	∗
	KIR2DL4	0.024	0.584	0.002	0.964
	KIR3DL1	0.206	∗∗∗	0.168	∗∗∗
	KIR3DL2	0.138	∗∗	0.106	∗
	KIR3DL3	0.029	0.508	0.017	0.712
	KIR2DS4	0.08	0.0634	0.058	0.217
Dendritic cell	HLA-DPB1	0.221	∗∗∗	0.193	∗∗∗
	HLA-DQB1	0.208	∗∗∗	0.185	∗∗∗
	HLA-DRA	0.257	∗∗∗	0.23	∗∗∗
	HLA-DPA1	0.264	∗∗∗	0.25	∗∗∗
	BDCA-1 (CD1C)	0.185	∗∗∗	0.175	∗∗∗
	BDCA-4 (NRP1)	0.218	∗∗∗	0.207	∗∗∗
	CD11c (ITGAX)	0.038	0.385	0.025	0.591
Th1	T-bet (TBX21)	0.093	∗	0.064	0.167
	STAT4	−0.002	0.956	−0.04	0.886
	STAT1	0.219	∗∗∗	0.208	∗∗∗
	IFN-g (IFNG)	0.053	0.22	0.034	0.461
	TNF-a (TNF)	0.062	0.152	0.043	0.358
Th2	GATA3	−0.212	∗∗∗	−0.165	∗∗∗
	STAT6	0.323	∗∗∗	0.31	∗∗∗
	STAT5A	0.139	∗∗	0.16	∗∗∗
	IL13	−0.059	0.17	−0.088	0.0588
Tfh	BCL6	−0.076	0.0793	−0.089	0.0570
	IL21	−0.072	0.0972	−0.09	0.0546
Th17	STAT3	0.237	∗∗∗	0.239	∗∗∗
	IL17A	−0.031	0.472	−0.029	0.532
Treg	FOXP3	−0.127	−0.143	∗∗	∗∗
	CCR8	0.04	0.361	0.028	0.552
	STAT5B	0.433	∗∗∗	0.417	∗∗∗
	TGFb (TGFB1)	−0.253	∗∗∗	−0.271	∗∗∗
T cell exhaustion	PD-1 (PDCD1)	0.036	0.404	0.016	0.733
	CTLA4	0.012	0.78	−0.004	0.925
	LAG3	0.017	0.694	0.009	0.855
	TIM-3 (HAVCR2)	0.355	∗∗∗	0.32	∗∗∗
	GZMB	−0.029	0.507	−0.066	0.157

^*∗*^ *P* < 0.05; ^*∗∗*^*P* < 0.01; ^*∗∗∗*^*P* < 0.001.

**Table 3:** The kinase, miRNA, and transcription factor target networks of ACE2 in KIRC (LinkedOmics).

Enriched category	Gene set	Leading edge number	*P* value
Kinase target	Kinase_LCK	29	0
	Kinase_LYN	30	0
	Kinase_SYK	16	0
	Kinase_JAK3	8	0
	Kinase_HCK	17	0
miRNA target	GTGCCAA, MIR-96	91	0
	TGCACTT, MIR-519 C, MIR-519 B, MIR-519A	140	0
	TGCACTG, MIR-148A, MIR-152, MIR-148B	97	0
	GTGCCTT, MIR-506	213	0
	TATTATA, MIR-374	105	0.002
Transcription factor target	V$IRF_Q6	104	0
	V$NFKB_Q6_01	57	0
	V$ELF1_Q6	84	0
	V$PEA3_Q6	81	0
	V$PU1_Q6	150	0

## Data Availability

The analyzed datasets generated during the study are available from the corresponding author on reasonable request.
